# Community science for mosquito surveillance in Canada: a toolkit for program design and implementation

**DOI:** 10.17269/s41997-025-01134-1

**Published:** 2025-11-23

**Authors:** Wendy Pons, Negar Elmieh, Atanu Sarkar, Angelo Armijos-Carrion, Tom Chapman, Amandeep Hans, Margaret Haworth-Brockman, Stefan Iwasawa, Victoria Ng, Jullia Santos, Jade Savage, Divya Zalawadia

**Affiliations:** 1 Conestoga College, Kitchener, ON Canada; 2 National Collaborating Centre for Environmental Health, Vancouver, BC Canada; 3 Memorial University of Newfoundland, St. John’s, NB Canada; 4 Windsor-Essex County Health Unit, Windsor, ON Canada; 5 National Collaborating Centre for Infectious Diseases, Winnipeg, MB Canada; 6 British Columbia Centre for Disease Control, Vancouver, BC Canada; 7 Public Health Agency of Canada, Toronto, ON Canada; 8 Bishop’s University, Sherbrooke, QC Canada

**Keywords:** Community, Mosquito control, Surveillance program, Citizen science, Communauté, Lutte contre les moustiques, Programme de surveillance, Science citoyenne

## Abstract

**Setting:**

Climate change in Canada has expanded suitable habitats for both native and invasive mosquitoes, heightening the need for more robust and adaptable surveillance systems. Traditional surveillance methods face significant logistical and geographical challenges, particularly in remote or underserved areas, emphasizing the importance of innovative strategies that complement existing approaches.

**Intervention:**

We developed a community-based mosquito surveillance toolkit, informed by a systematic review and validated by a multidisciplinary expert group. The toolkit includes a structured guide for developing a community science project, including how to design an effective mosquito surveillance program through data collection, volunteer engagement, and evaluation. It also features a quick reference guide and a 90-s animated video to facilitate understanding and engagement.

**Outcome:**

The toolkit was introduced at a national community meeting, with 69 participants bringing together public health professionals, educators, scientists, and community leaders from across Canada in February 2025. Participants expressed enthusiasm for using the toolkit to enhance local monitoring efforts, and discussions at the meeting helped identify key opportunities and challenges for adoption.

**Implications:**

Organizations and public health authorities now have a clear, evidence-based guide to designing, implementing, and evaluating community-driven initiatives. This toolkit not only helps ensure that volunteer participation is meaningful and effective, but it also enhances the capacity to respond to emerging challenges in vector control, laying the groundwork for sustainable, community-driven surveillance efforts.

## Résumé

**Contexte:**

Le changement climatique au Canada a élargi les habitats adaptés à la fois pour les moustiques indigènes et envahissants, renforçant la nécessité de disposer de systèmes de surveillance plus robustes et plus adaptables. Les méthodes de surveillance traditionnelles font face à d'importants défis logistiques et géographiques, en particulier dans les zones éloignées ou mal desservies, soulignant l'importance de stratégies innovantes qui complètent les approches existantes.

**Intervention:**

Nous avons développé une boîte à outils de surveillance des moustiques basée sur la communauté, élaborée à partir d'une revue systématique et validée par un groupe d'experts multidisciplinaires. La boîte à outils comprend un guide structuré pour développer un projet de science communautaire, incluant comment concevoir un programme efficace de surveillance des moustiques à travers la collecte de données, l'engagement des bénévoles et l'évaluation. Elle comprend également un guide de référence rapide et un vidéo animée de 90 secondes pour faciliter la compréhension et l'engagement.

**Résultat:**

La boîte à outils de surveillance a été présentée lors d'une réunion communautaire nationale, réunissant 69 participants, dont des professionnels de la santé publique, des éducateurs, des scientifiques et des dirigeants communautaires de tout le Canada en février 2025. Les participants ont exprimé leur enthousiasme à utiliser la boîte à outils pour améliorer les efforts de suivi locaux, et les discussions lors de la réunion ont permis d'identifier les principales opportunités et les défis liés à son adoption.

**Implications:**

Les organisations et les autorités de santé publique disposent désormais d'un guide clair et fondé sur des preuves pour concevoir, mettre en œuvre et évaluer des initiatives communautaires. Cette boîte à outils permet non seulement de garantir que la participation des bénévoles est significative et efficace, mais elle renforce également la capacité à répondre aux défis émergents dans le contrôle des vecteurs, jetant ainsi les bases d’efforts de surveillance communautaire durables.

## Introduction

Climate change has significantly altered ecological dynamics globally, and in Canada. Rising temperatures and changing precipitation patterns have altered the habitats suitable for mosquito species (Ludwig et al., 2019; Ng et al., 2019). Species that were previously confined to more temperate regions are now migrating northward, introducing new vectors to these areas capable of transmitting diseases like West Nile virus (Reisen, 2013), Zika virus (Robert et al., 2019), and dengue fever (Nguyen et al., 2020). International trade and transportation networks in Canada provide additional ways for the accidental introduction of exotic mosquito species into Canada (Ogden et al., 2019).

Canada’s vast geography presents unique challenges for conventional mosquito monitoring (Ludwig et al., 2019; Ogden et al., 2019). While routine mosquito surveillance is often concentrated in populated regions where human health risk is highest, limited access to remote and rural areas, often inaccessible or difficult to survey regularly (Kulkarni et al., 2015), can hinder the timely detection of emerging mosquito-borne threats before they spread to more populated zones, potentially resulting in data gaps (Hoshi et al., 2019).

Existing mosquito surveillance programs in Canada are limited by constrained resources, both in terms of funding and personnel. Traditional surveillance methods, such as CO_2_ baited traps, light traps, and professional field sampling, can be resource-intensive and may overlook species with limited attraction to traps, and their effectiveness can vary depending on environmental conditions (Mata et al., 2020). These limitations affect the frequency and scope of monitoring activities (Forest-Berard et al., 2021). Additionally, there is often a lack of real-time data sharing between different jurisdictions and agencies, leading to fragmented surveillance efforts (Nguyen et al., 2023). Integrating data from various sources is crucial for identifying trends and responding promptly to potential outbreaks. Without comprehensive and coordinated surveillance, public health responses may be delayed and the risk misrepresented, reducing the effectiveness of interventions.

Community science, also known as citizen science, involves the active engagement of non-professional scientists in the scientific process (Gharaibeh et al., 2019). Participation can range from data collection and analysis to the dissemination of findings. By involving community members directly, community science empowers individuals to contribute to research that addresses local and global challenges. Community science democratizes scientific research, fostering a sense of ownership and responsibility among participants (Atias et al., 2023). In mosquito surveillance, community science can enable the public to assist in monitoring mosquito populations, identifying species, and detecting pathogens (Sousa, Craig, et al., 2022; Sousa, Fricker, et al., 2022a, 2022b). This collaborative approach enhances the spatial and temporal coverage of surveillance efforts, especially in areas that are difficult to access through conventional methods, or in areas lacking government programs. Moreover, it promotes public education and awareness about mosquito-borne diseases and environmental health (Ghosh et al., 2013). This empowerment can lead to more responsive and effective public health interventions, as communities become active stakeholders in the surveillance and control of mosquito populations.

While community science offers numerous advantages, it also faces challenges. Data quality can vary depending on participant training and adherence to protocols, project longevity may be limited by volunteer availability and interest, and coverage can be uneven across communities (Callaghan et al., 2022). Data from these projects often does not provide a true representation of the mosquito species present; however, it can be appropriate for estimating species abundance or presence, indicating where more formal surveillance is needed and identifying early patterns in the data. The intent of community science data is not to replace existing formal mosquito surveillance activities but rather to supplement and enhance existing systems (Callaghan et al., 2022). Several successful community science programs highlight the potential of public involvement in environmental surveillance (Dickinson et al., 2012). The eTick program in Canada engages individuals in actively participating in public health surveillance by submitting photographs of ticks they encounter (eTick, 2024). Experts promptly identify the species and provide informative feedback, which contributes to a dynamic, real-time map of tick distributions. This tool not only supports monitoring the potential spread of tick-borne diseases, such as Lyme disease, but also raises awareness among participants by educating them about tick species and associated health risks. By engaging the public in data collection, eTick strengthens surveillance efforts while fostering community involvement in public health.

Similarly, iNaturalist is a global online community where users share observations of biodiversity, including insects such as mosquitoes. Participants upload photographs and location data, which are verified by a network of experts and citizen scientists. The aggregated data from iNaturalist has been utilized in numerous scientific studies, improving our understanding of species distributions and ecological changes (Ipin et al., 2023). Adapting these models to mosquito surveillance in Canada could significantly enhance data collection efforts. By leveraging technology and community engagement, these programs demonstrate how citizen contributions can fill gaps in traditional surveillance methods, leading to more informed public health strategies.

Designing a successful collaborative framework in community science projects involve strategic partnerships among diverse organizations. Clear objectives, well-defined roles, and inclusive participation are essential components that leverage the strengths of each partner (King et al., 2021). Health departments, environmental groups, and research teams are typically involved in these collaborations. Health departments provide expertise in disease surveillance and vector management and control, contributing critical knowledge to monitoring efforts. Environmental groups offer insights into habitats and ecosystems, along with strategies for engaging communities in environmental stewardship. Research teams bring scientific rigour, data analysis capabilities, and technical training support, ensuring that the project’s methodologies are sound and effective. Each organization plays a specific and vital role, creating a comprehensive approach to community science initiatives (Costa et al., 2021; King et al., 2021).

Partnerships in community science projects yield significant benefits (Costa et al., 2021; King et al., 2021). Collaborative efforts maximize resources by sharing equipment and technical expertise, thereby reducing operational costs. They expand the geographical reach of data collection, especially in remote or hard-to-access areas. Partnerships also enhance community trust and credibility, motivating greater community participation. Moreover, interdisciplinary approaches stimulate innovation, enriching strategies to address public health concerns through diverse perspectives.

Community science projects focused on mosquito surveillance have been successfully carried out in several parts of Canada. Notable examples include a study in Newfoundland and Labrador, where citizen scientists effectively collected mosquito samples from remote regions in a cost-efficient manner (Hollett et al., 2025). In Windsor, Ontario, the TIMO-CS study, developed through a collaboration between the Public Health Agency of Canada and Let’s Talk Science, was designed to complement existing surveillance activities and identify *Aedes albopictus* (tiger mosquitoes), a species not typically found in that region (Government of Canada, 2024). Other Canadian efforts include a citizen science campaign in British Columbia led by entomologist Dan Peach, which encouraged residents to send in mosquito specimens for analysis (Labbé & Kelly, 2022), and a summer-long trapping initiative in New Brunswick, where volunteers contributed to mosquito research by monitoring local populations (CBC News, 2024). While these projects demonstrate the potential of community involvement in mosquito surveillance, those involved in these initiatives have identified gaps in the lack of a centralized resource to support others interested in launching similar programs. Such a resource would help future users avoid common challenges, promote consistency, and encourage broader participation in community-driven science.

The primary objective of this project was to develop a comprehensive toolkit designed to address the following critical gaps in mosquito surveillance: (1) empower community science initiatives by standardizing data collection protocols; (2) enhance collaboration among health departments, environmental groups, and research teams; and (3) integrate community-collected data into formal surveillance systems. By providing a unified framework, this toolkit aims to improve surveillance coverage, foster public health education, and build sustainable partnerships. Ultimately, our goal is to support the creation of community science initiatives that enhance formal surveillance programs and strengthen responses to mosquito-borne disease risks, ensuring that communities are better equipped to protect themselves.

## Our intervention

The development of the Community Science Toolkit for Mosquito Surveillance followed a structured process comprising two main phases. In the first phase, a systematic review was conducted that focused on community/citizen science strategies for mosquito surveillance. This initial analysis allowed the identification of relevant approaches for habitat mapping, species detection and characterization, pathogen identification, data management, evaluation, and participant training. Specific results of this systematic review are presented in a forthcoming publication.

The second phase, and the focus of this paper, was the development of three key resources designed to support mosquito surveillance and community science initiatives. This project brought together professionals from the field with expertise in entomology, epidemiology, public health, and community science. The primary researchers (WP, NE, AS) conceptualized the idea for the toolkit and an Expert Advisory Group (EAG) provided subject matter expertise on content and guidance on the relevance, clarity, and feasibility of the proposed contents. The EAG critically reviewed its structure and engaged in discussions that addressed key aspects of its implementation. The EAG paid particular attention to language level and literacy for all users of the documents, data quality, and consideration of the practical application of the toolkit in communities.

The toolkit was developed to meet the needs of various groups, such as health professionals, environmental organizations, local institutions, educational institutions, and community groups interested in developing a community science project. The toolkit’s development was also informed by a review of other mosquito/community science/public health toolkits from North America, which helped shape our approach, though none was directly comparable.

**Fig. 1:**
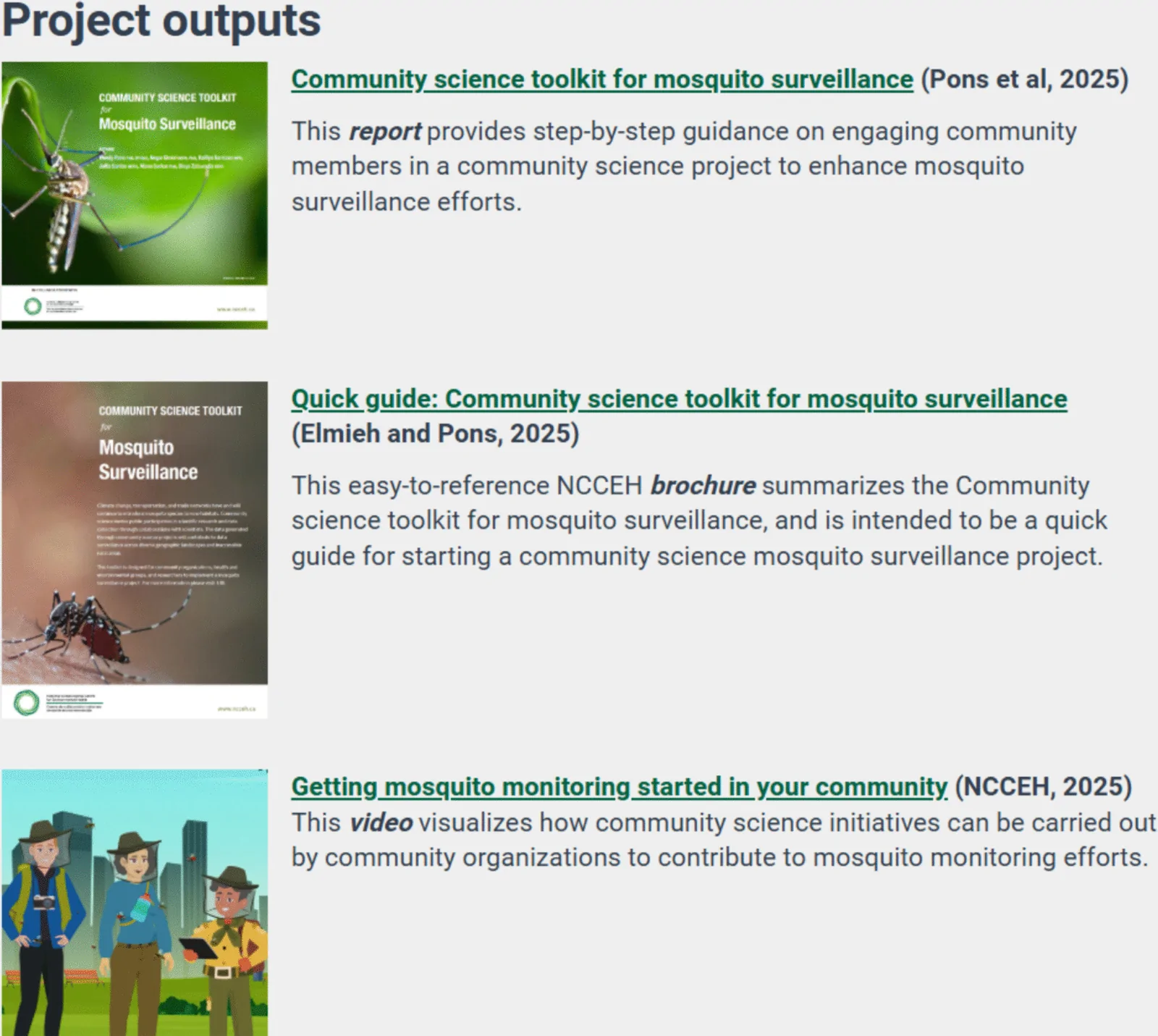
Three resources to support mosquito surveillance community science initiatives, available on the NCCEH website: https://ncceh.ca/resources/subject-guides/community-science-approaches-mosquito-surveillance

The toolkit contains three resources: a primary resource, which is a comprehensive 54-page document, offering in-depth guidance for organizations and citizen scientists. To complement this, a four-page quick guide document provides a concise summary of the primary resource highlighting the key elements, and a 90-s animated video promotes the other two resources and demonstrates their practical applications in an engaging and visually appealing format (displayed in Fig. 1).

**Fig. 2:**
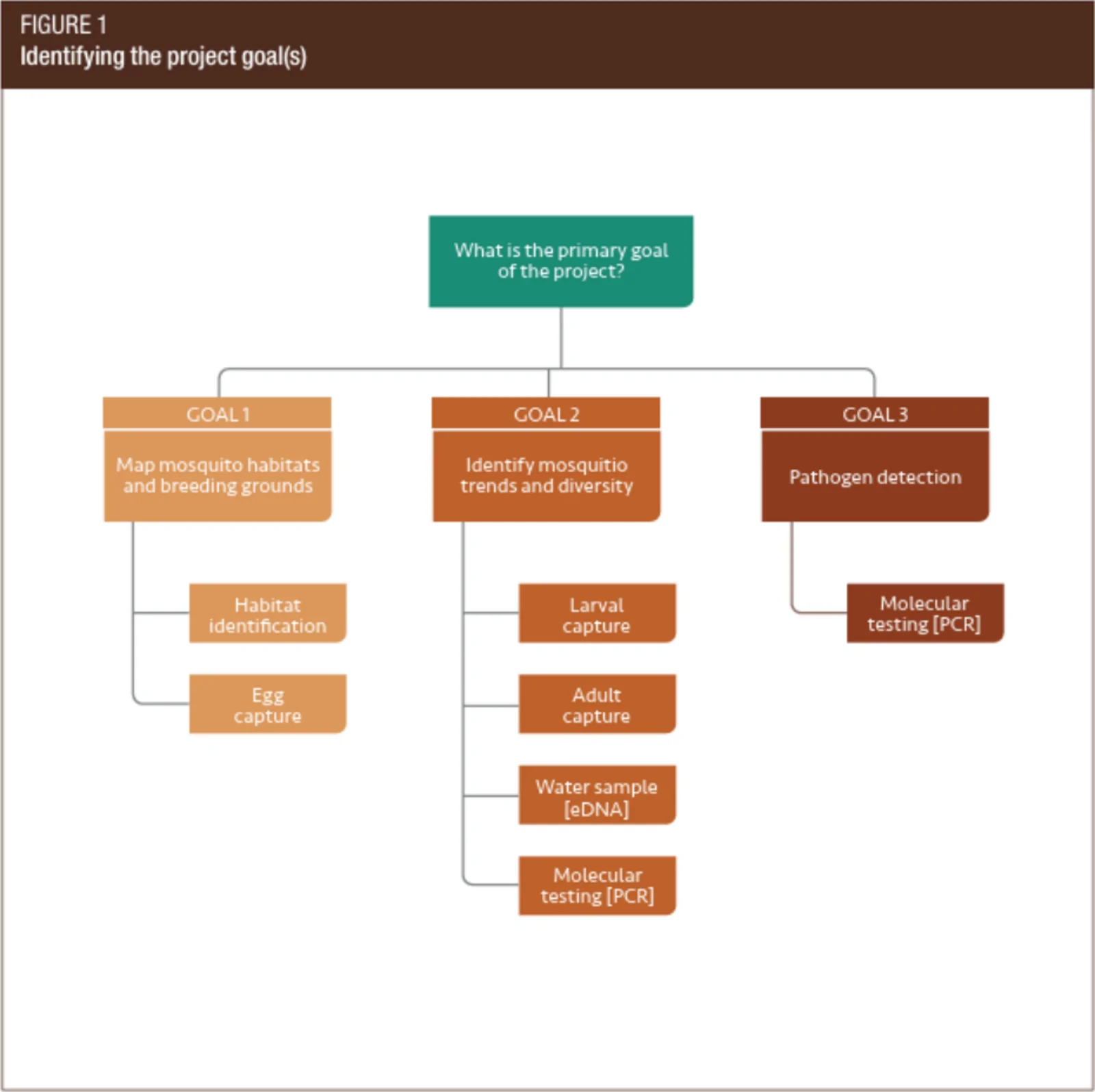
A resource from the toolkit designed to help users determine their initial approach to project development

**Fig. 3:**
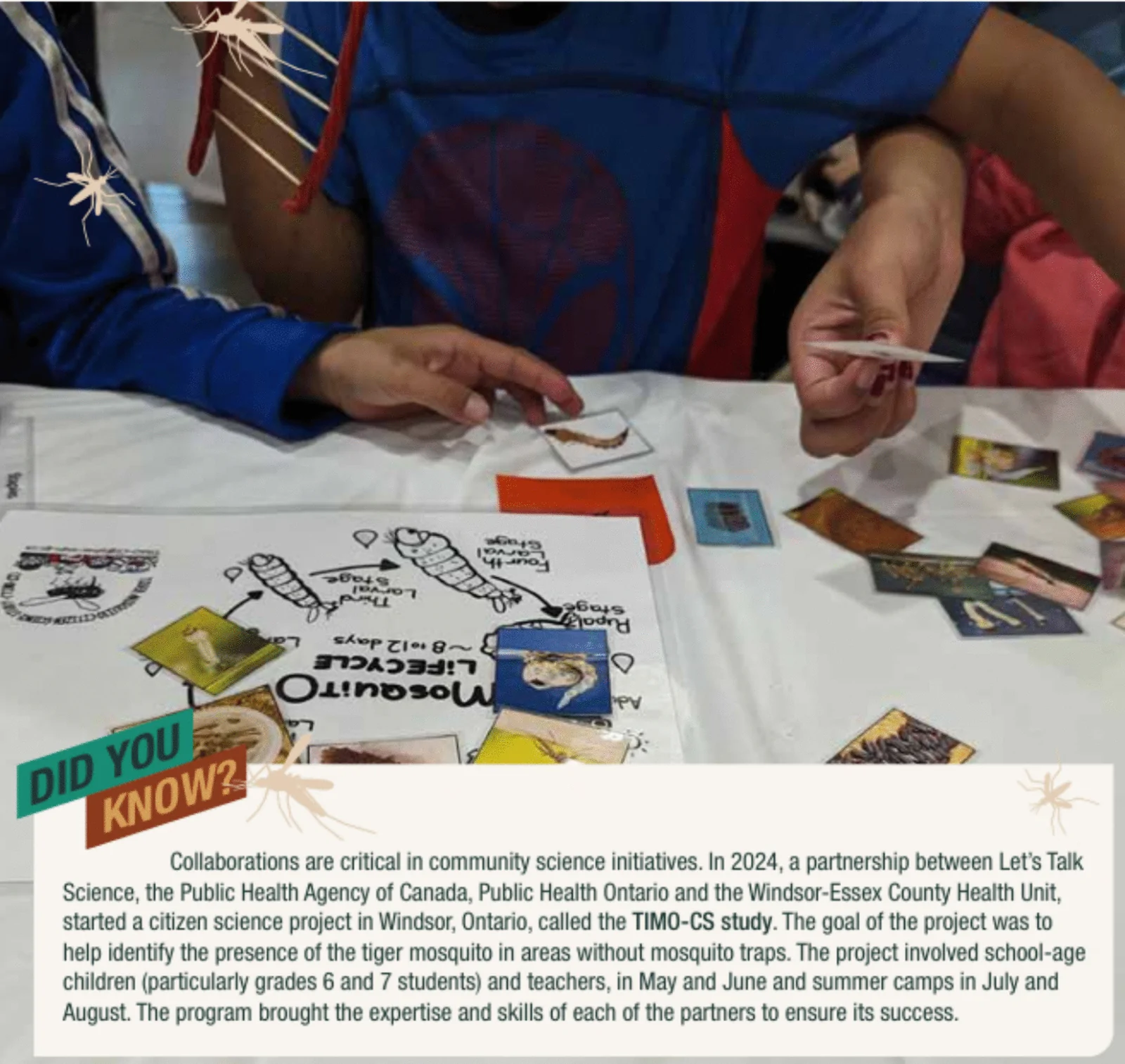
An example from the toolkit showcasing successful projects and ideas to guide users in developing their own initiatives

**Fig. 4:**
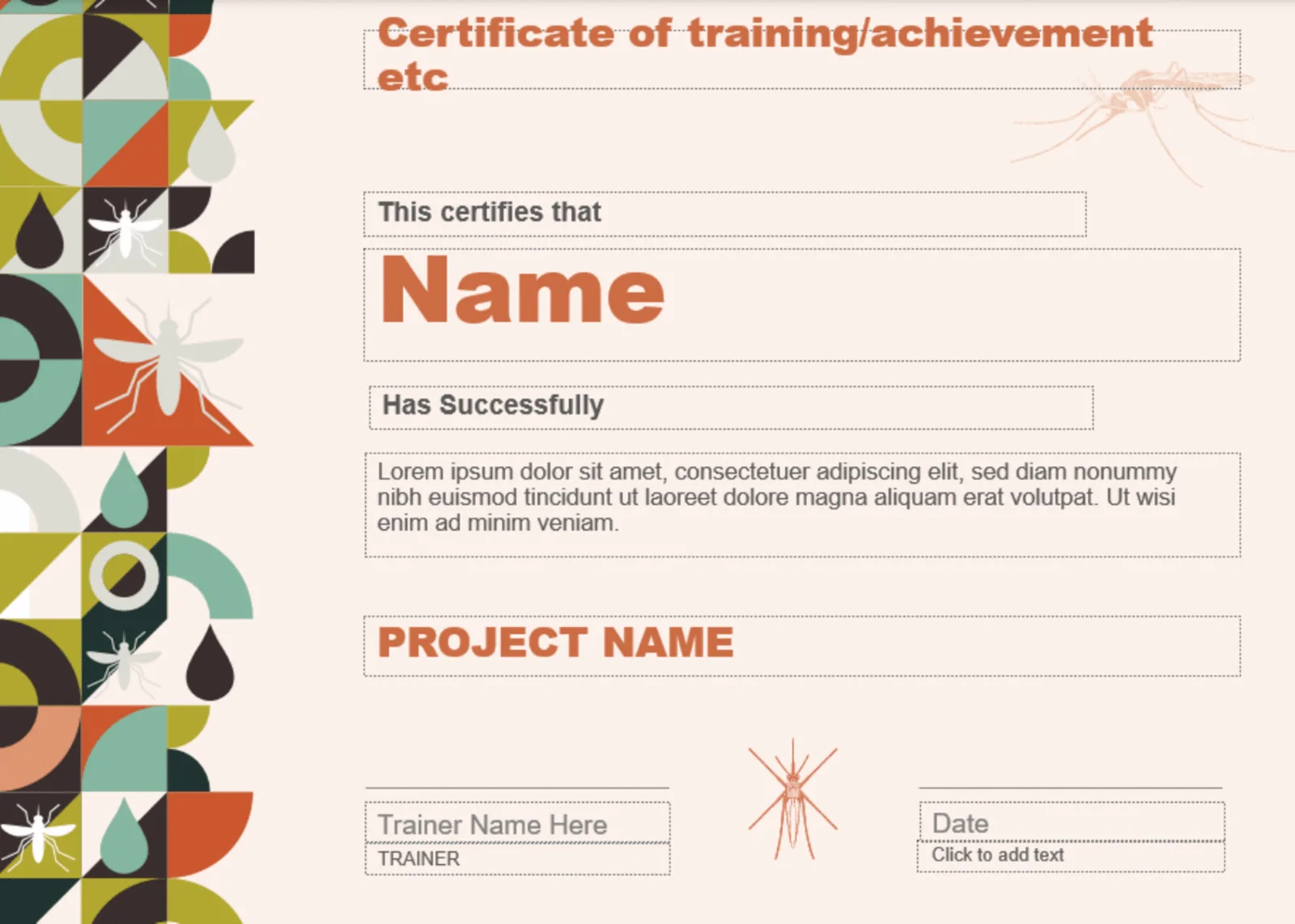
A fillable certificate available in the toolkit that users can modify to fit the needs of their own program

The primary resource includes three sections: (1) designing a community science project; (2) community involvement and outreach; (3) evaluating a community science project. Because community science seeks to accommodate a range of needs and contexts, the guide provides information on integrating traditional field methods and advanced molecular techniques, such as PCR and environmental DNA analysis (one example is shown in Fig. 2). The toolkit also provides quality assurance criteria, recommendations for participant training, guidance for informed decision-making, references to other community science projects that users can use to guide their own project (as seen in Fig. 3), directives for integration into existing surveillance frameworks, and resources like fillable certificates that users can adapt to fit their own needs (as seen in Fig. 4). Feedback from the EAG led to numerous revisions to balance scientific precision and practical applicability, resulting in a guide for enhancing community participation in mosquito surveillance. All toolkit materials are hosted on the National Collaborating Centre for Environmental Health (NCCEH) website, ensuring easy access for users (https://ncceh.ca/resources/subject-guides/community-science-approaches-mosquito-surveillance). To support users, an email contact is available for feedback, inquiries, or assistance in implementing the toolkit within their programs. The written documents are offered in both French and English.

## Outcomes

We launched the Community Science Toolkit for Mosquito Surveillance, at a 90-min virtual national community meeting that brought together 69 potential users of the toolkit from across Canada. Invitations were shared with targeted organizations through mosquito network groups and the NCCEH listserve, reflecting a broad and relevant audience. This event included a panel presentation introducing the toolkit, provided an overview of its features, followed by breakout group discussions where participants could ask questions, explore potential applications, and spark dialogue among diverse stakeholders invested in community science and public health. These discussions focused on the potential applications of the toolkit and its usefulness in potential projects. Participants also provided feedback and suggestions on the toolkit for improvements. The meeting also facilitated opportunities for networking and collaboration. Attendees included public health professionalsfrom provincial and municipal health units, educators from universities, environmental scientists, and community organizers, reflecting a diverse group of stakeholders with relevant expertise and interest in community science and mosquito surveillance who might not typically work together. By combining a comprehensive toolkit with a collaborative rollout event, we introduced a valuable resource. Since the meeting, we are aware that two groups are actively using the toolkit. One is implementing a project in the summer of 2025, and another is planning a project for 2026. These early adopters illustrate the potential of the toolkit.

## Discussion

This work underscores the potential of community-based mosquito surveillance initiatives to address public health challenges, particularly as changing climates and expanding mosquito ranges increase the need for effective monitoring (Palmer et al., 2017). When funding for government mosquito programs declines or is insufficient, community science offers a cost-effective and adaptable approach to bridge critical gaps, especially in hard-to-reach or underserved areas. However, it should not be viewed as a substitute for formal surveillance, nor should governments scale back their efforts and rely solely on community science to shoulder the responsibility for surveillance.

With proper training, feedback, and support, volunteers can collect valuable data to support meaningful surveillance. However, the successful integration of community-collected data into existing systems requires standardized protocols and harmonized methodologies (Garamszegi et al., 2023). Government and public health policies must prioritize resources for training, effective communication, and infrastructure to ensure the sustainability of these initiatives over time. When volunteers understand the importance of their contributions, witness tangible outcomes, and feel supported by professionals and community leaders, their engagement tends to deepen (Ngo et al., 2023). With careful planning, it is possible to build a foundation that balances scientific rigour with real-world practicality (Bonney et al., 2009).

The toolkit developed in this project was designed with these principles in mind. It has the potential to enhance the resilience and cost-effectiveness of mosquito surveillance programs by complementing traditional methods. The toolkit provides standardized protocols, training guidelines, and integration strategies that can be tailored to local conditions, ultimately enabling community participants to contribute more effectively to ongoing surveillance efforts.

## Conclusion

As climate change alters ecosystems and expands species ranges across Canada, collaborative community approaches, supported by this toolkit, will become increasingly important for mosquito surveillance. While there will always be challenges in maintaining interest, ensuring quality, aligning different kinds of data, and securing sufficient resources, there is reason for optimism. With thoughtful design, clear communication, and strong partnerships, community science can complement traditional surveillance methods, contributing to more robust and responsive public health strategies (Sousa et al., 2020). In this way, the newly developed toolkit is designed to support efforts to not only help detect emerging threats more quickly and efficiently but also empower citizens to play an active role in protecting their own communities from the potential impacts of climate change.

## “So what” section

What are the innovations in this policy or program?

- This project offers a detailed framework for involving communities in mosquito surveillance efforts across Canada, to address critical gaps in conventional monitoring.
- Organizations and public health authorities now have a comprehensive guide to designing, implementing, and evaluating community-driven initiatives.
- This framework helps ensure that volunteer participation is meaningful and effective, and expands surveillance to regions that are hard to monitor with traditional methods.
- In practical terms, this means being able to supplement surveillance efforts of mosquito species in Canada or detect invasive mosquito species and emerging diseases earlier and more efficiently, reinforcing decision-making with reliable, community-sourced information.

What are the burning research questions for this innovation?

- The next phase of this work focuses on evaluating how the toolkit supports the planning and delivery of community-based mosquito surveillance projects.
- Key questions include: Which components of the toolkit are most effective or need improvement? And how well does it adapt to diverse settings and populations?
- Further evaluation will provide critical insights into its real-world impact, helping to refine the resource and inform future public health investments in citizen science and vector-borne disease prevention.

## Data Availability

Toolkit is available on a public website: https://ncceh.ca/resources/subject-guides/community-science-approaches-mosquito-surveillance.
